# Tohoku Medical Megabank Brain Magnetic Resonance Imaging Study: Rationale, Design, and Background

**DOI:** 10.31662/jmaj.2022-0220

**Published:** 2023-06-30

**Authors:** Makiko Taira, Shunji Mugikura, Naoko Mori, Atsushi Hozawa, Tomo Saito, Tomohiro Nakamura, Hideyasu Kiyomoto, Tadao Kobayashi, Soichi Ogishima, Fuji Nagami, Akira Uruno, Ritsuko Shimizu, Tomoko Kobayashi, Jun Yasuda, Shigeo Kure, Miyuki Sakurai, Ikuko N. Motoike, Kazuki Kumada, Naoki Nakaya, Taku Obara, Kentaro Oba, Atsushi Sekiguchi, Benjamin Thyreau, Tatsushi Mutoh, Yuji Takano, Mitsunari Abe, Norihide Maikusa, Yasuko Tatewaki, Yasuyuki Taki, Nobuo Yaegashi, Hiroaki Tomita, Kengo Kinoshita, Shinichi Kuriyama, Nobuo Fuse, Masayuki Yamamoto

**Affiliations:** 1 Tohoku Medical Megabank Organization, Tohoku University, Sendai, Japan; 2 Graduate School of Medicine, Tohoku University, Sendai, Japan; 3 Tohoku University Hospital, Tohoku University, Sendai, Japan; 4 Advanced Research Center for Innovations in Next-Generation Medicine, Tohoku University, Sendai, Japan; 5 Miyagi Cancer Center, Natori, Japan; 6 Miyagi Children’s Hospital, Sendai, Japan; 7 Graduate School of Information Sciences, Tohoku University, Sendai, Japan; 8 Institute of Development, Aging and Cancer, Tohoku University, Sendai, Japan; 9 Integrative Brain Imaging Center, National Center of Neurology and Psychiatry, Tokyo, Japan; 10 University of Human Environments, Matsuyama, Japan; 11 Graduate School of Medicine, Fukushima Medical University, Fukushima, Japan; 12 Graduate School of Art and Science, University of Tokyo, Tokyo, Japan; 13 International Research Institute of Disaster Science, Tohoku University, Sendai, Japan; 14 The United Centers for Advanced Research and Translational Medicine, Tohoku University, Sendai, Japan

**Keywords:** The Tohoku Medical Megabank Brain Magnetic Resonance Imaging Study (TMM Brain MRI Study), TMM Community-Based Cohort Study (TMM CommCohort Study), TMM Birth and Three-Generation Cohort Study (TMM BirThree Cohort Study), neuroimaging, neuropsychological assessments, cognitive impairment

## Abstract

The Tohoku Medical Megabank Brain Magnetic Resonance Imaging Study (TMM Brain MRI Study) was established to collect multimodal information through neuroimaging and neuropsychological assessments to evaluate the cognitive function and mental health of residents who experienced the Great East Japan Earthquake (GEJE) and associated tsunami. The study also aimed to promote advances in personalized healthcare and medicine related to mental health and cognitive function among the general population. We recruited participants for the first (baseline) survey starting in July 2014, enrolling individuals who were participating in either the TMM Community-Based Cohort Study (TMM CommCohort Study) or the TMM Birth and Three-Generation Cohort Study (TMM BirThree Cohort Study). We collected multiple magnetic resonance imaging (MRI) sequences, including 3D T1-weighted sequences, magnetic resonance angiography (MRA), diffusion tensor imaging (DTI), pseudo-continuous arterial spin labeling (pCASL), and three-dimensional fluid-attenuated inversion recovery (FLAIR) sequences. To assess neuropsychological status, we used both questionnaire- and interview-based rating scales. The former assessments included the Tri-axial Coping Scale, Impact of Event Scale in Japanese, Profile of Mood States, and 15-item Depression, Anxiety, and Stress Scale, whereas the latter assessments included the Mini-Mental State Examination, Japanese version. A total of 12,164 individuals were recruited for the first (baseline) survey, including those unable to complete all assessments. In parallel, we returned the MRI results to the participants and subsequently shared the MRI data through the TMM Biobank. At present, the second (first follow-up) survey of the study started in October 2019 is underway. In this study, we established a large and comprehensive database that included robust neuroimaging data as well as psychological and cognitive assessment data. In combination with genomic and omics data already contained in the TMM Biobank database, these data could provide new insights into the relationships of pathological processes with neuropsychological disorders, including age-related cognitive impairment.

## Introduction

In March 2011, the Great East Japan Earthquake (GEJE) and related tsunami led to the devastation of a large area of northeastern Japan. Studies have investigated the massive impact of the GEJE and the ensuing tsunami on the people and infrastructure of the prefectures located along the Pacific coast in that region ^(1), (2), (3), (4)^. A total of 15,899 people died, and 2,526 individuals were never found ^(5), (6)^. To understand the causes and mechanisms of chronic diseases that increased in prevalence following the disaster as well as those due to aging, the Tohoku Medical Megabank (TMM) was established with support from the Japanese government ^(1)^. The TMM then conducted two large longitudinal cohort studies: the Community-Based Cohort Study (TMM CommCohort Study) ^(2)^ and the Birth and Three-Generation Cohort Study (TMM BirThree Cohort Study) ^(3), (4)^. We have strategically set up these two cohorts with the aim of monitoring the damage to the health status of residents in the GEJE-affected areas. The TMM CommCohort Study involved a relatively elderly population, whereas the BirThree Cohort Study included pregnant women and children. Both groups suffered from the earthquake and tsunami more seriously than the others; thus, we aimed to follow up the long-term damage to them. We also planned to reduce the cohort size while potentiating the power to detect the gene-environment relationship of diseases by using the family relationship in the BirThree Cohort Study. In addition, we expected that the combination of the two cohorts will allow us to use one as a discovery cohort and the other as a variation cohort, which will enhance the capture of rare variants with high accuracy ^(3)^. As the governing body over these studies, we have established the TMM Organization ^(1), (7)^.

In recent years, prospective, large-scale cohort studies using neuroimaging and neuropsychiatric evaluation methods have provided a comprehensive understanding of the precursors and potential risks of diseases in currently asymptomatic individuals ^(8)^. Understanding of neuropsychiatric disease processes can be enhanced through neuroimaging (e.g., magnetic resonance imaging [MRI]) and rating scales to evaluate psychological processes; both techniques are highly versatile and provide reliable data for longitudinal observational studies. Furthermore, longitudinal observational studies that include successive measurements using questionnaire instruments prevent bias owing to variability in clinicians’ assessments of risk factors and the incidence of clinical events ^(9)^. The use of neuroimaging and rating scales in cross-sectional studies (such as hospital-based cohort studies) may also provide results that are less susceptible to recall bias ^(10)^. Therefore, we established the Program of Health Surveillance on the Brain and Psychological State with MRI in 2014. Here, the program is referred to as the TMM Brain MRI Study, “the study,” or “our study.”

## Aims of the TMM Brain MRI Study

While some individuals who experience a disaster are mentally and emotionally affected, others remain relatively unaffected ^(11)^. Among the affected individuals, some recover quickly, whereas others experience long-term impacts; there are several and diverse factors that differentiate these groups ^(11)^. Thus, the priority of the preparatory committee of the TMM project was to conduct an in-depth investigation of neuropsychological factors related to the population’s responses to the GEJE and related tsunami.

**Figure 1.**
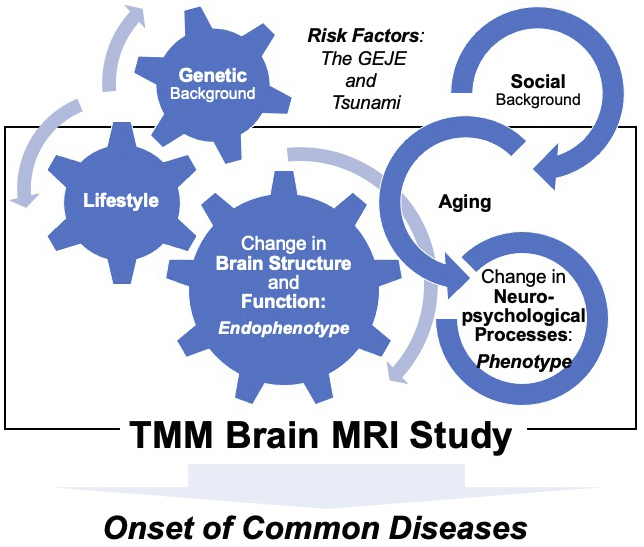
Conceptual framework of the Tohoku Medical Megabank (TMM) Brain MRI Study. The TMM Brain MRI Study was designed to assess the risk factors for the onset of multifactorial neurological diseases as well as the possible influence of the Great East Japan Earthquake (GEJE) and tsunami. By examining the interaction of genetic, biological, social, and environmental factors in the development of manifestations, such as brain abnormalities (endophenotype) and clinical characteristics (phenotype) of common aging-associated diseases, the study could pave the way to the detection of prodromal features in older adults that precede age-related, systemic, or neurological deterioration.

The American Psychiatric Association’s Diagnostic and Statistical Manual of Mental Disorders, 5^th^ edition (DSM-5), is one of the most widely used classification systems by mental health professionals worldwide ^(12), (13)^. The DSM-5 defines a mental disorder as a significant disturbance in an individual’s cognition, emotion regulation, or behavior that reflects dysfunction in the psychological, biological, or developmental processes underlying mental functioning ^(11), (12)^. The World Health Organization (WHO) reported that mental health is determined by socioeconomic, biological, and environmental factors ^(14), (15), (16), (17)^. These concepts emphasize the necessity of a comprehensive investigative framework, which was carefully considered by the study (Figure 1).

Thus, one of the primary goals of this study was to collect relevant, multimodal structural brain imaging and psychological profiling data so as to grasp the changes in the mental health of regional residents affected by the GEJE and tsunami.

Another major goal of this study was to promote advances in personalized healthcare and medicine for the treatment of cognitive and mental disorders. Therefore, the study also aimed to establish a comprehensive and strategic foundation that could lead to drug discovery and development for the prevention and treatment of neurodegenerative diseases in later life, in line with urgent demands (Figure 1). The number of patients with dementia worldwide currently exceeds 55 million, and by 2030, it is expected to reach approximately 78 million ^(18)^. Prospective cohort-based epidemiologic studies have been recognized as one of the most effective approaches to understanding dementia and clarifying its risk factors and causes ^(19), (20)^. This is why all data with cognitive screening of ostensibly healthy elderly participants enrolled in the study are tied to the wealth of genetic and environmental information collected from the two primary cohort studies.

## Recruitment Procedure for the TMM Brain MRI Study

**Figure 2.**
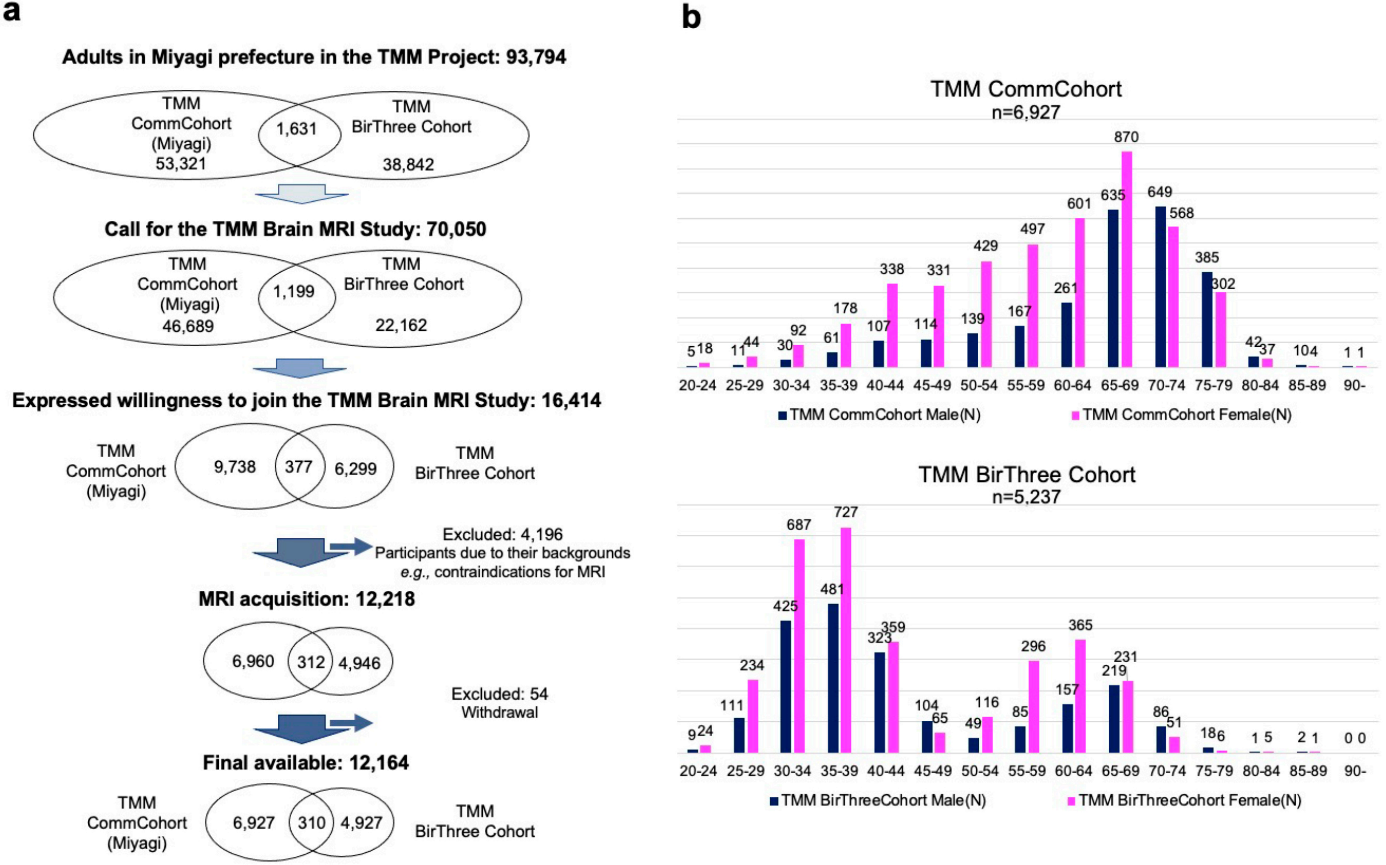
Recruitment flow chart of the TMM Brain MRI Study. a. Participants in the TMM Brain MRI Study were recruited from those who had already participated in two cohort studies, the TMM Community-based Cohort Study (TMM CommCohort Study) and the TMM Birth and Three Generations Cohort Study (TMM BirThree Cohort Study), and have lived in Miyagi Prefecture. b. This figure presents the age and gender distribution of participants in the TMM CommCohort and TMM BirThree Cohort. Note that the age distributions are sharply different. Participants who participated in both the TMM CommCohort Study and TMM BirThree Cohort Study were counted as participants in the TMM BirThree Cohort Study.

Because the major goal of the study was to examine the relationship between health status and changes in the brain structure of residents in GEJE-affected areas, study recruitment was designed to assemble a broad and representative sample of such individuals. Thus, we enrolled individuals already participating in the TMM CommCohort Study and the TMM BirThree Cohort Study (Figure 2a). The TMM CommCohort Study included adult men and women aged over 20 years from Miyagi and Iwate Prefectures ^(1), (2)^. The TMM BirThree Cohort Study included three generations within families, including pregnant women, their future children and (fetuses), and extended family members ^(1), (3), (4)^.

The TMM Brain MRI Study included adult participants from both cohorts but only among individuals living in Miyagi Prefecture. Of the 93,794 possible participants ^(1), (2), (3), (4)^, we contacted 70,050 individuals by mailing an invitation letter or through community outreach health promotion activities conducted by the TMM (Figure 2a). Of these individuals, 16,414 were willing to participate in the study, and 12,164 completed the consent form and took part in the first (baseline) survey.

The important demographic characteristics of the participants are presented in Figure 2b. To avoid overcounting, those who participated in both cohort studies were counted only in the TMM BirThree Cohort Study. In almost every age group, the number of women was larger than that of men. An important characteristic of the study is its very wide age distribution, ranging from 20 to over 90 years old. This wide distribution was due to the strategic design that included participants from both primary cohorts. Specifically, most of the participants aged over 65 years were recruited from the TMM CommCohort Study, and most of those aged below 50 years were recruited from the TMM BirThree Cohort Study. Although recruiting such a broad sample was challenging, it was crucial to establish a comprehensive reference group for MRI data.

## Timeline of the TMM Brain MRI Study

**Figure 3.**
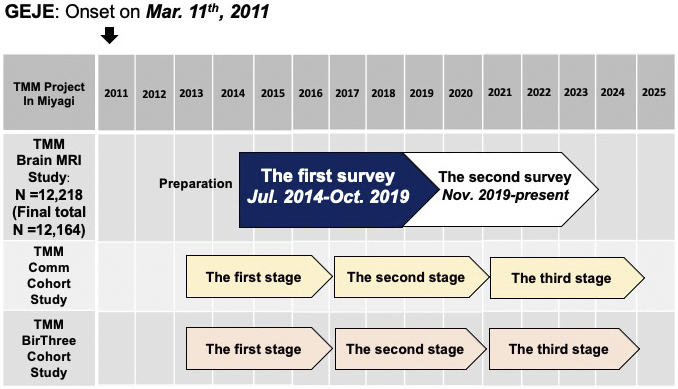
Timeline of the TMM Brain MRI Study and its relation to the TMM CommCohort Study and TMM BirThree Cohort Study. The timeline of the TMM Brain MRI Study is presented in comparison with the timeline of the TMM CommCohort Study and the TMM BirThree Cohort Study. The first (baseline) survey of the TMM Brain MRI Study was conducted from July 2014 to Oct. 2019. It was conducted until late October 2019. The second (first follow-up) study began on the last business day of October 2019. Please note that the start and end dates for the TMM Brain MRI Study are later than the start and end dates for the two cohort studies.

A timeline of the TMM Brain MRI Study is presented in Figure 3, along with those for the two primary cohort studies (TMM CommCohort Study and BirThree Cohort Study) that served as the basis for participant recruitment in the study.

The two cohort studies have been so far conducted in three stages ^(1), (2), (3), (4)^. The participants underwent extensive assessments of lifestyle characteristics and health surveys in the first stage from 2013 to 2017 and the second stage from 2017 to 2021 or are now undergoing the third stage, along with blood and urine tests ^(21)^ that resulted in the large-scale acquisition ^(22)^ of genomic and omics data ^(23), (24), (25), (26), (27), (28)^. Reflecting these backgrounds, our study has thus far involved two surveys. The first (baseline) survey took place from July 2014 to October 2019. This period overlapped with the first and second stages of the two primary cohort studies (Figure 3). The second (first follow-up) survey was initiated at the end of October 2019. Now, it is ongoing and overlaps with the second and third stages of the primary cohort studies. Thus, it is available to connect participants’ data in our study with their data at several visits to the primary cohort studies.

Regardless of which side of the registration to the two primary cohorts, all adult individuals without MRI contraindications, such as metal implants, were considered eligible for our study; such individuals who provided informed consent were enrolled. The participants in our study needed to visit the Sendai Community Support Center separately from their visits to the primary cohort studies (Figure 3).

## Data Management System for MRI Data Acquired from the TMM Brain MRI Study

**Figure 4.**
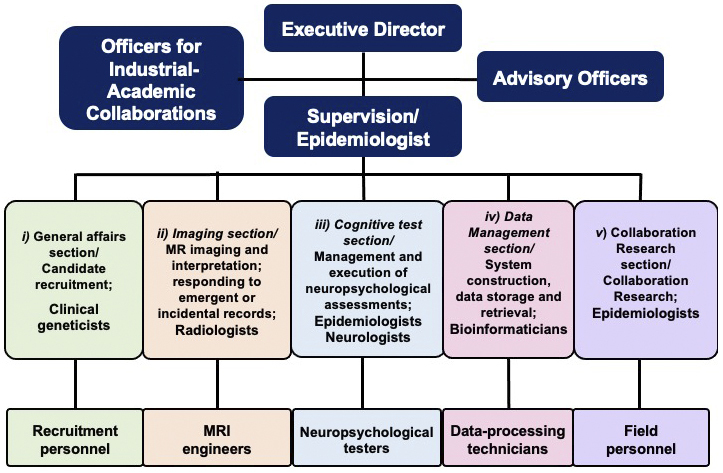
Organization, leadership, and staffing of the TMM Brain MRI Study. Experts from a variety of disciplines bring together their academic knowledge and experience to manage the TMM Brain MRI Study through five special teams. Experts include clinical geneticists, radiologists, epidemiologists, bioinformaticians, and neurologists.

The TMM Brain MRI Study was designed in a multilayer fashion that fits within the Biobank management system. We aimed to store the data in the Biobank and share it with wide-ranging users. To efficiently conduct neuroimaging and neuropsychological assessments, or to implement data storage and robust sharing, we established five sections to oversee the operation of the study (Figure 4). These comprised of *i*) a General Management section responsible for the recruitment of candidates; *ii*) an Imaging section responsible for MRI scans, including their conduct, processing, interpretation, and response to significant incidental findings; *iii*) a Cognitive Assessment section responsible for the management and administration of cognitive assessments; *iv*) a Data Management section responsible for system construction, data storage, and retrieval; and *v*) a Collaborative Research section that handled and accelerated the use of MRI data through the TMM Biobank. Specialists, including MRI engineers, recruitment personnel, individuals performing cognitive assessment, and data processing technicians, actively participated in the five sections. A neurologist specializing in dementia coordinated the activities of the whole section.

## Overview of Experimental Procedure of the TMM Brain MRI Study

**Figure 5.**
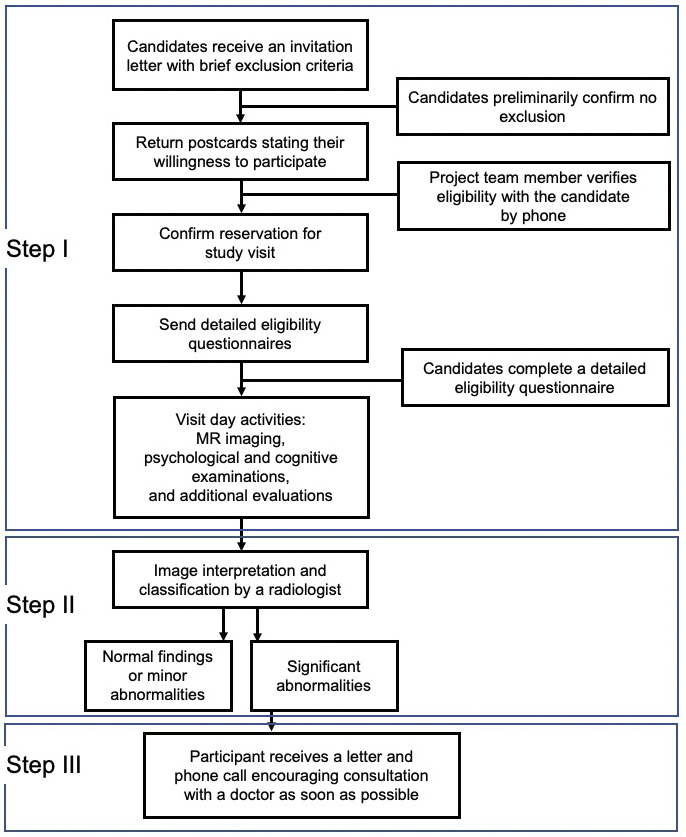
Steps from recruitment to return of the MRI results. The flowchart presents the implementation of this TMM Brain MRI Study in three phases. Phase I is from candidate recruitment to actual participation, and Phases II and III are from recruitment to return of the results. Note that Phase III indicates that a letter may be sent to the participant recommending immediate medical review and consultation at a medical institution based on findings incidentally obtained on the brain images that require medical attention.

Figure 5 presents an overview of the experimental procedure for the study. As presented in the flow chart, the procedure has three important characteristics. First, the TMM staff and participants communicated (Phase I). Second, we returned the MRI results to all participants according to the principles of medical ethics (Phase II). Third, we prioritized the communication of emergency results to the participants (Phase III).

Recruitment of the participants involved sequential steps (Figure 5). First, a recruitment guide was mailed to potential participants. This guide contained general information regarding the study, basic eligibility-related questions, and a postcard that interested candidates could return to indicate their willingness to participate. After receiving the postcard, a member of the recruiting team made a phone call to the candidates who responded to explain the study in more detail and review the answers to the screening questions. To determine MRI contraindications, more detailed questions were asked; if direct dialog with a doctor was considered necessary, a doctor made a follow-up call to the candidate. For each potential participant who passed this preliminary assessment, the team member reserved a date and time for a visit to the study center that was convenient for the candidate. Figure 5 also presents high-level information regarding the visit day, image interpretation, and return of the imaging results, which are described in detail below.

## Visit the Schedule of Each Participant

**Figure 6.**
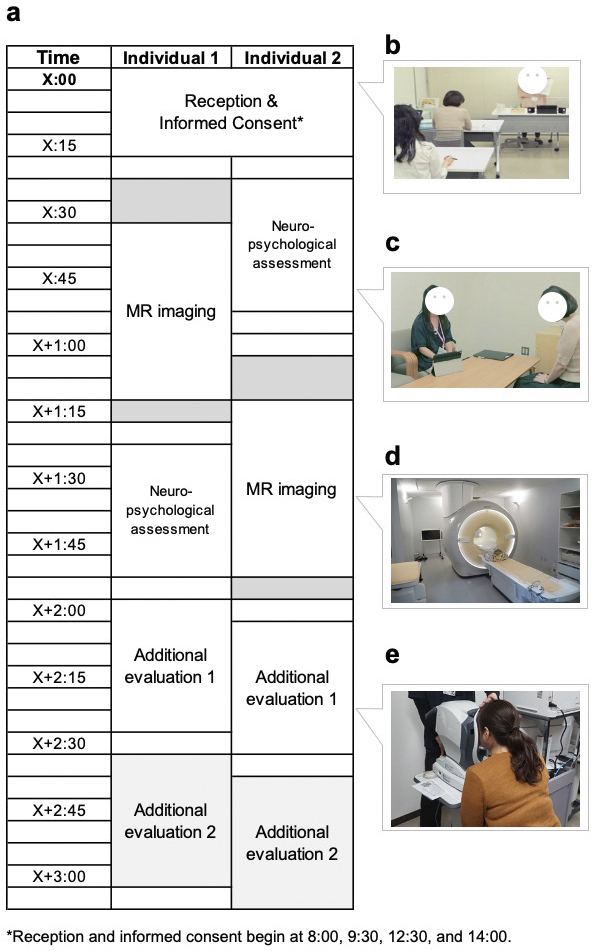
Procedures experienced by participants on the day of the visit for each data acquisition. a. The procedure for the two participants who have their opening reception at the same time of day is divided into 15-min segments, and they alternate between each examination to avoid wasting time. b. Informed consent is provided at the same time, but participants are asked to sign individually to express their consent to participate in the testing. c. Neuropsychological evaluation is conducted by trained testers. d. The total image acquisition time per participant during MR imaging vary depending on the imaging conditions at each time point. e. Additional evaluations, such as retinal imaging, is performed on the same visit date.

Figure 6 presents a representative visit schedule for the participants. Neuropsychological data were obtained before or after MRI acquisition based on practical considerations. Upon arrival, the study procedures were thoroughly reviewed with each participant, and their consent to participate in the study was obtained. The consent process followed the principles of medical ethics, specifically candidates’ right to participate (or not) based on a full understanding of the concepts and content of the consent form (i.e., not just “agreement”); individuals who refused to participate after receiving an explanation were excluded. Each applicant could also ask questions and request explanations until satisfied. This is an important concept about rights derived from medical ethics.

## The Procedure for Brain MRI Scans

**Figure 7.**
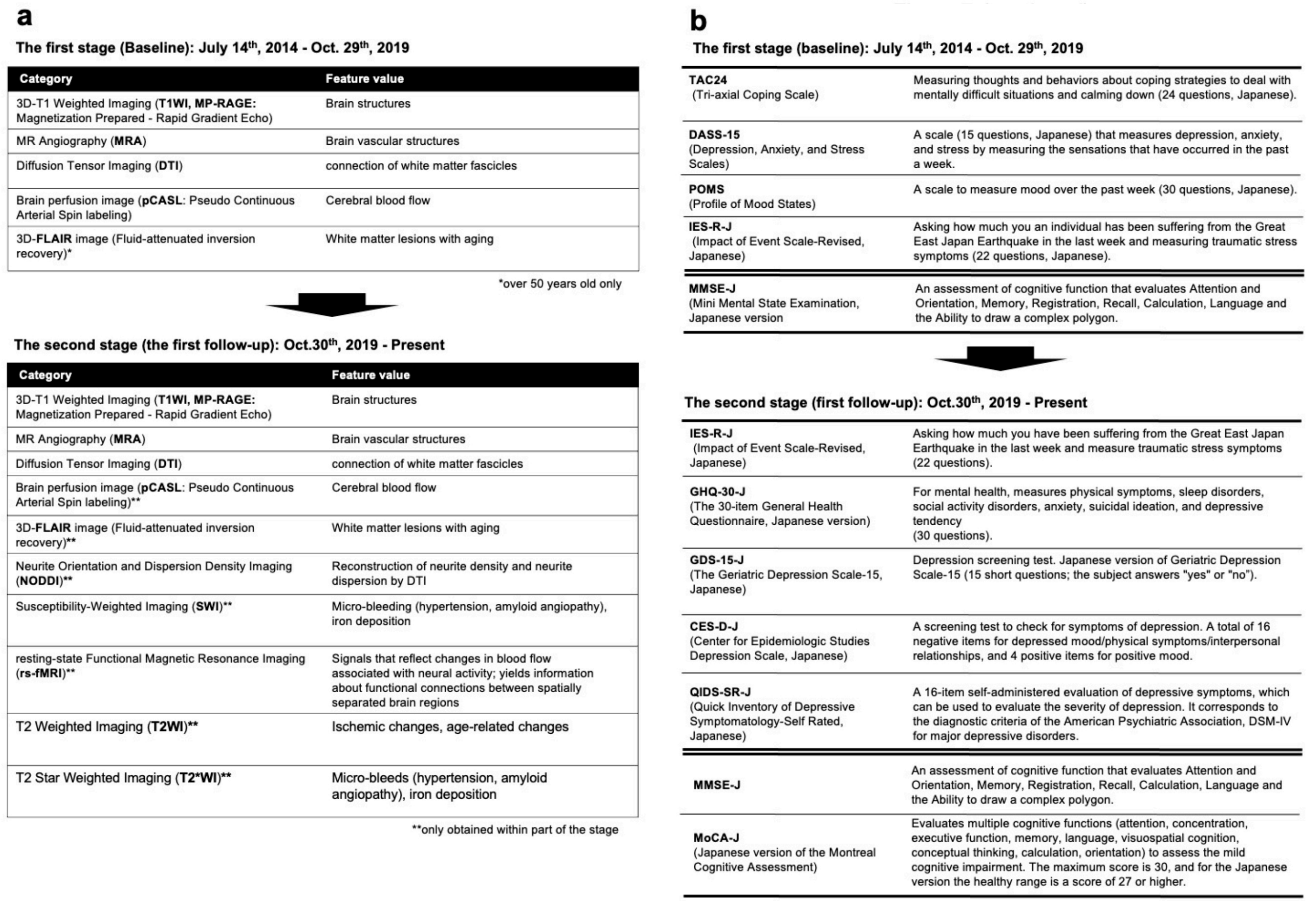
MRI sequences and neuropsychological assessments during the first (baseline) and second (first follow-up) surveys. a. The figure presents MRI sequences in the first (baseline) and second (first follow-up) surveys. The first survey (baseline) included 3D T1-weighted imaging (T1W1; MP-RAGE), magnetic resonance angiography (MRA), diffusion tensor imaging (DTI), brain perfusion imaging (pCASL), and 3D-FLAIR sequences. The second (first follow-up) survey has added neurite orientation and dispersion density imaging (NODDI), susceptibility-weighted imaging (SWI), resting-state functional magnetic resonance imaging (rs-fMRI), T2-weighted imaging (T2WI), and T2*-weighted imaging (T2*WI). b. The figure presents questionnaires and on-site (interview) assessments in the first (baseline) and second (first follow-up) surveys at the top and bottom tables, respectively. Questionnaires are shown above the double line, whereas on-site assessments are shown below the line. In both surveys, the participants completed questionnaires assessing stress, psychiatric, personality, and neuropsychological factors at home and brought the completed forms with them on the day of the visit. On-site neuropsychological testing using the MMSE-J has been administered by trained testers. In the second (first follow-up) survey, the Montreal Cognitive Assessment, Japanese version (MoCA-J), is now administered to all participants.

Two MRI scanners (Philips Medical System Ingenia 3.0 Tesla) were dedicated to the study. These scanners had identical settings, a 32-channel head coil, and a full-body coil. An image acquisition protocol was designed to obtain data from the entire brain. The MRI sequences in the first survey with the aspects of the brain structure or the assessed function are presented in Figure 7a, Table 1, and Table 2, along with the numbers of participants who underwent each sequence. We used the following MRI sequences: *i*) 3D T1-weighted imaging (T1WI) using the magnetization-prepared rapid gradient echo (MP-RAGE) ^(29)^ method without a contrast agent to examine brain structure; *ii*) magnetic resonance angiography (MRA) to characterize brain vasculature ^(30)^; *iii*) diffusion tensor imaging (DTI) ^(31)^ to delineate white matter tracts; *iv*) pseudo-continuous arterial spin labeling (pCASL) ^(32)^, a type of perfusion imaging, to identify cerebral blood flow patterns; and* v*) three-dimensional fluid-attenuated inversion recovery (3D-FLAIR) ^(33)^ imaging to indicate the presence and characteristics of white matter hyperintensities. All participants underwent 3D T1WI and MRA, but not all participants underwent DTI and pCASL. 3D FLAIR was performed only in a subset of participants aged over 50 years ^(34)^, resulting in 2,428 images in the first (baseline) survey.

**Table 1.** MRI Protocol at the First (Baseline) Survey.

Sequence (Data type)	Measured property and commentary	Sequence parameters											
		Representative images (number of participants)	Acquisition & slice	TR (ms)	TE (ms)	Inversion time (ms)	SENSE: AP RL	b-value (s/mm^2^)	FOV (mm)	Matrix	Slice thickness (mm)	FA (degrees)	Acquisition time (s)
T1-weighted imaging (T1WI) ^(29)^	Brain structures obtained by magnetization prepared-rapid gradient echo (MP-RAGE)	12,164	3D Sagittal	11	5.2	1068.3	2 2.5	N/A	256	368 × 368	0.7	8	319
Magnetic resonance angiography (MRA) ^(30)^	Brain vascular structure	12,050	3D Axial	25	3.5	0	1(FH) 3	N/A	200	384 × 249	1.1	18	247
Diffusion tensor imaging (DTI) ^(31)^	Interaction or connection of white matter fascicles in the brain	10,443	2D Axial	7173	83	0	2.5 N/A	800 (32)^2^	224	112 × 112	2	90	318
Pseudo-continuous arterial spin-labeling (pCASL)^(32)^	Cerebral blood flow^1^	10,461	2D Axial	4023	11	0	2.1 N/A	N/A	240	64 × 64	7/1	90	247
Fluid-attenuated inversion recovery (FLAIR) ^(33)^	White matter lesions (collected in those aged >50 years only)	2,428	3D Sagittal	4800	276	1650	3 3.2	N/A	256	212 × 212	1.2	90	264

Notes: Parenthesized numerical references correspond to bibliographic citations elsewhere in the manuscript. TR: repetition time, TE: echo time, SENSE: sensitivity encoding, FOV: field of view, FA: flip angle. D: Dimension, AP: anterior and posterior directions, RL: right and left directions, FH: foot and head direction N/A: Not applicable TR, TE, and Inversion time could be variable according to the slice angle. ^1^ Post-labeling delay: 1,800 ms ^2^ The number in parentheses is the number of diffusion directions.

**Table 2.** MRI Protocol in the Second (First Follow-Up) Survey.

Sequence (Data type)	Measured property and commentary	Sequence parameters										
		Acquisition & slice	TR (ms)	TE (ms)	Inversion time (ms)	SENSE: AP RL	b-value (s/mm^2^)	FOV (mm)	Matrix	Slice thickness (mm)	FA (degrees)	Acquisition time (s)
T1-weighted imaging (T1WI) ^(29)^	Brain structures obtained by magnetization prepared-rapid gradient echo (MP-RAGE)	3D Sagittal	11	5.2	1068.3	2 2.5	N/A	256	368 × 368	0.7	8	319
Magnetic resonance angiography (MRA) ^(30)^	Brain vascular structure	3D Axial	25	3.5	0	1(FH) 3	N/A	200	384 × 249	1.1	18	247
Diffusion tensor imaging (DTI) ^(31)^	Interaction or connection of white matter fascicles in the brain	2D Axial	7173	83	0	2.5 N/A	800	224	112 × 112	2	90	318
Pseudo-continuous arterial spin-labeling (pCASL) ^(32)^	Cerebral blood flow^1,^	2D Axial	4148	11	0	2.1 N/A	N/A	240	64 × 64	7/1	90	247
Fluid-attenuated inversion recovery (FLAIR) ^(33)^	White matter lesions	3D Sagittal	4800	276	1650	3 3.2	N/A	256	212 × 212	1.2	90	264
Neurite orientation dispersion and density imaging (NODDI) ^(35)^	Microstructure of neurites (i.e., axons and dendrites)	2D Axial	7278	111	N/A	3 N/A	711 (30)^2^ 2855 (60)^2^	230	112 × 109	2.5	90	944
Resting state-functional-MR imaging (Rs-fMRI): Protocol 1 ^(36)^	Functional connectivity	2D Axial	3000	30	N/A	3 N/A	N/A	224	76 × 73	3	90	372
Rs-fMRI: Protocol 2 ^(37)^	Functional connectivity	2D Axial	2500	30	N/A	N/A N/A	N/A	212	64 × 63	3.2	80	612
T2-weighted imaging	White matter lesions	2D Axial	4134	100	N/A	N/A 2	N/A	230 × 221	384 × 269	5/1	90	157
T2*-weighted imaging ^(38)^	Cerebral microbleeds	2D Axial	664	18	N/A	N/A 2	N/A	230 × 217	304 × 229	5/1	18	78
Susceptibility-weighted imaging (SWI) ^(38)^	Cerebral microbleeds	3D Axial	31	7.2	N/A	(FH) 1.5 3	N/A	230 × 189	384 × 316	2	17	181

Notes: Parenthesized numerical references correspond to bibliographic citations elsewhere in the manuscript. TR: repetition time, TE: echo time, SENSE: sensitivity encoding, FOV: field of view, FA: flip angle. D: Dimension, AP: anterior and posterior directions, RL: right and left directions, FH: foot and head direction N/A: Not applicable TR, TE, and Inversion time could be variable according to the slice angle. ^1^ Post-labeling delay: 1,800 ms ^2^ The number in the parenthesis is the number of diffusion directions.

In addition, neurite orientation and dispersion density imaging (NODDI) ^(35)^, resting-state functional MRI (rs-fMRI) ^(36), (37)^, T2-weighted imaging, T2*-weighted imaging ^(38)^, and susceptibility-weighted imaging (SWI) ^(38)^ were added to the second survey (first follow-up) (Figure 7a and Table 2). NODDI is a method for quantifying free diffusion in the brain using a theoretical mathematical model called the Watson distribution, which assumes that the microstructure of the brain can be divided into three compartments: restricted diffusion within cells, bounded diffusion between cells, and free diffusion of the cerebrospinal fluid component. This method is useful for assessing neurite degeneration as it reflects the distribution and synaptic connections of neurites (dendrites and axons). rs-fMRI is used to measure signals that reflect blood flow changes associated with neural activity and yield information regarding functional connectivity between spatially separated brain regions. Although these are valuable data that can be used to detect early diagnostic changes in the brain regions of the healthy population, it takes slightly more time to make these data public. We will finish the second MR imaging and will clean up data within a few years before data sharing.

## Assessments of the Neuropsychological Status of the Participants

A variety of neuropsychological assessments were conducted in the study. The rationale for reinforcing the measures of mental health data was twofold: first, to assess the impact of the GEJE and tsunami on the mental or emotional status of residents, and second, to probe into early diagnostic markers of cognitive impairment linked to MRI scans.

To this end, in the first (baseline) survey, we primarily used four questionnaires and one in-person examination (Figure 7b and Table 3). To evaluate psychological characteristics, we mailed the questionnaires to the participants 1 week before their visit and requested that they bring the completed versions on their visit day (Figure 5). These questionnaires were selected to assess the participants’ coping skills and the presence of mood disorders, such as depression or anxiety. The Tri-axial Coping Scale (TAC24-J) ^(39)^ measures internal states and behaviors related to challenging circumstances, whereas the Depression, Anxiety, and Stress Scales, 15-item version (DASS-15) ^(40)^, measures the indicated affective states. The Profile of Mood States (POMS) ^(41)^ assesses broad mood states. The Impact of Event Scale-Revised (IES-R) ^(42)^ in Japanese ^(43)^ assesses subjective distress caused by traumatic events. Of the 12,164 participants in the first (baseline) survey, 12,156, 12,156, 12,144, and 12,153 provided data on the TAC24, DASS-15, POMS, and IES-R, respectively. In the second (first follow-up) survey, psychological evaluations for anxiety (GHQ-30-J) ^(44)^, senile depressive status (GDS-15-J) ^(45)^, depression (CES-D) ^(46)^(QIDS-SR-J) ^(45)^, and traumatic stress reactions (QIDS-SR-J) ^(47)^ were added from the perspective of differentiating between dementia and various senile psychological states (Figure 7b).

**Table 3.** Psychological and Cognitive Instruments in the First (Baseline) Survey.

Data acquisition method	Instrument name*	Measured phenomena	Number of items/subscales, if applicable	Number of participants
Questionnaire	TAC24 ^(39)^ (Tri-axial Coping Scale)	This scale measures coping, defined as “an ever-changing cognitive-behavioral effort made to meet specific external and internal demands that are burdensome or have been rated as exceeding all personal resources,” by assessing participants’ thoughts, behaviors, and strategies for dealing with mentally difficult situations.	24 items/8 subscales	12,156
	DASS-15 ^(40)^ (Depression, Anxiety, and Stress Scales)	A scale that measures symptoms of depression, anxiety, and stress over the past week.	15 items/3 subscales	12,156
	POMS ^(41)^ (Profile of Mood States)	This scale measures six different dimensions of mood swings (tension or anxiety, anger or hostility, vigor or activity, fatigue or inertia, depression or dejection, and confusion or bewilderment) over a period.	30 items/6 subscales	12,144
	IES-R-J ^(42), (43)^ (Impact of Event Scale-Revised, Japanese)	The IES-R is a self-report measure that assesses subjective distress caused by traumatic events. The items directly correspond to 14 of the 17 symptoms of posttraumatic stress disorder (PTSD) according to the Diagnostic and Statistical Manual of Mental Disorders, 4^th^ edition (DSM-IV). The IES-R contains seven additional items related to the hyperarousal symptoms of PTSD. Respondents are asked to identify a specific stressful life event (i.e., the Great East Japan Earthquake) and then indicate how much they were distressed or bothered by the difficulty listed during the past 7 days.	22 items/3 subscales	12,153
Interview	MMSE-J ^(56), (57)^ (Mini-Mental State Examination, Japanese version)	An assessment of cognitive function that evaluates attention and orientation, memory, registration, recall, calculation, language, and ability to draw a complex polygon.	11 items	3,610

Notes: Parenthesized numerical references correspond to bibliographic citations elsewhere in the manuscript. *: Representative psychological and cognitive instruments are shown.

## Return of MRI Results

**Figure 8.**
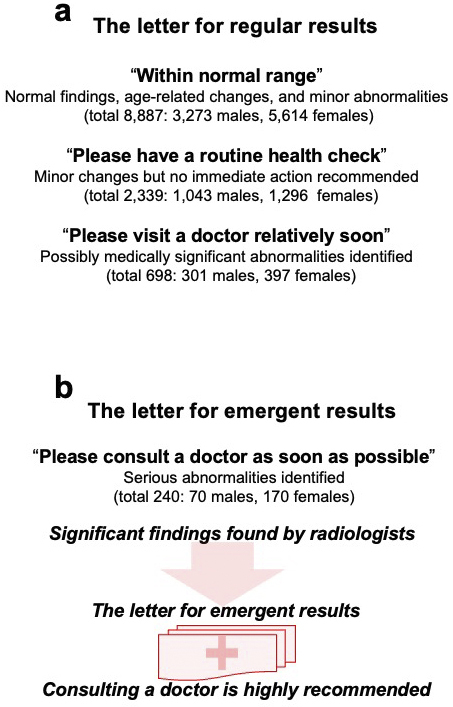
Return of the MRI results. TMM radiologists reviewed MRI scans and categorized abnormalities. Thereafter, participants are sent one of two types of letters communicating the scan findings, depending on the significance of the abnormalities. For both letters, the total number and sex distribution of participants are presented, along with the recommended follow-up. a. This is the content for the letter about regular results (including descriptions for each subcategory: normal, mild abnormalities, or noteworthy but nonemergent findings). b. This is the content for the letter about emergent findings.

MRI scans obtained during the visit were deliberated and classified by TMM radiologists. The system and procedure are presented in Figure 4 and 5, respectively. We informed the participants of the results through a letter; the content of a prototype letter for regular results is presented in Figure 8a. In this letter, we classified regular findings into three categories: normal, minor changes, and abnormal but nonurgent. Normal MRI results were labeled as “within the normal range”; this was the most common outcome (total 8,887: 3,273 men and 5,614 women). When minor changes were found, but the changes did not require immediate action, we sent a letter stating “minor changes were found, please have a routine health check” (total 2,339: 1,043 men and 1,296 women). If possibly medically significant but nonurgent abnormalities were found, the letter stated “Please visit a doctor relatively soon” (total 698: 301 men and 397 women).

If serious abnormalities were identified, we informed the participants of the results through a different letter; the content of a prototype letter for emergent results is presented in Figure 8b. This letter stated “Please consult a doctor as soon as possible.” We also called the participants (a total of 240: 70 men and 170 women). Details of the findings and procedures for this situation will be described separately.

## Pipeline for Sharing MRI Data

MRI data and other types of data are maintained in a supercomputer ^(22)^. The data have different security levels and are, therefore, maintained in different supercomputer units. The TMM supercomputer contained three units. Unit A is a public area, and individuals can access this area *via* L2/Internet. Contrarily, Unit B is a restricted area for collaborative research with academic or industrial partners outside the TMM; it enables data sharing. Unit C is a restricted area for researchers inside the TMM; it enables internal data evaluation. The security models used by the TMM have been previously described ^(48)^.

**Figure 9.**
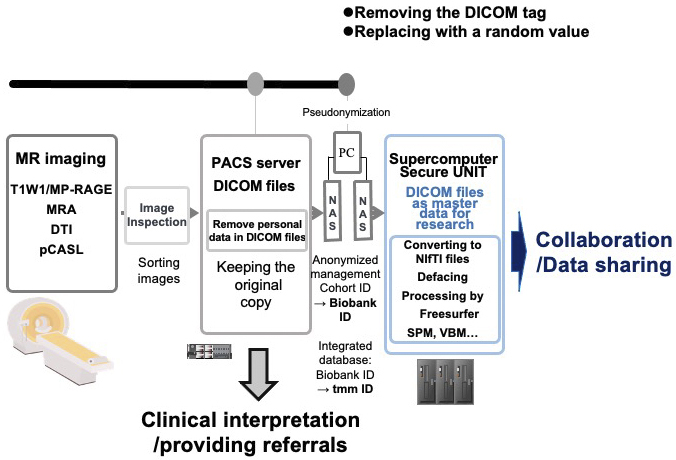
Pipeline for sharing MRI data. The processing scheme/flow from MRI data acquisition to distribution and sharing is shown: raw images in the DICOM format are transferred to the PACS server through a closed network. To share these data for research purposes, they are transferred to a standalone PC for pseudonymization *via* a USB hard disk drive (HDD) after removing personal information. Furthermore, to transfer the data to the supercomputer, the data after the pseudonymization process is exported to another USB HDD for further processing and research use.

Figure 9 presents the data management scheme and data flow. In the first step, images from various sequences (T1W1/MP-RAGE, MRA, DTI, and pCASL) were obtained. These images were examined by MR engineers and radiologists to ensure adequate data quality. For acceptable images, raw data in the Digital Imaging and Communications in Medicine (DICOM) ^(49)^ format were transferred to a Picture Archiving and Communication System (PACS) ^(50)^ server through a closed network. This step ensures the security of the data as raw image data contain some personal information, e.g., participant ID, month of birth, and sex.

To share these data for research purposes, we exported them by removing and replacing personal data in the DICOM files with a random value and transferred them to a standalone PC for pseudonymization using a USB hard disk drive (HDD). After the pseudonymization, processed DICOM data were exported to another USB HDD for transport to the supercomputer to enable further processing and research use. The DICOM data are the master data used by researchers; the original images maintained in the PACS server are used only for interpretation by physicians and not for research purposes. This arrangement (“firewall”) protects participants’ privacy. DICOM is a popular format for research use, but we also prepared files in the Neuroimaging Informatics Technology Initiative (NIfTI) ^(51)^ format, which is another popular format for research.

In an additional step to protect participant privacy, we routinely performed defacing using FreeSurfer ^(52)^. The defaced data and some feature values obtained by FreeSurfer can be widely used by researchers. Notably, for the convenience of users, we recalculated and distributed some types of processed data, such as statistical parametric mapping (SPM) ^(53)^ and voxel-based morphometry (VBM) ^(54)^ information.

## Preliminary Results Obtained from Neuroimaging and Neuropsychological Assessments

We have conducted two preliminary analyses based on the data obtained to date.

**Figure 10.**
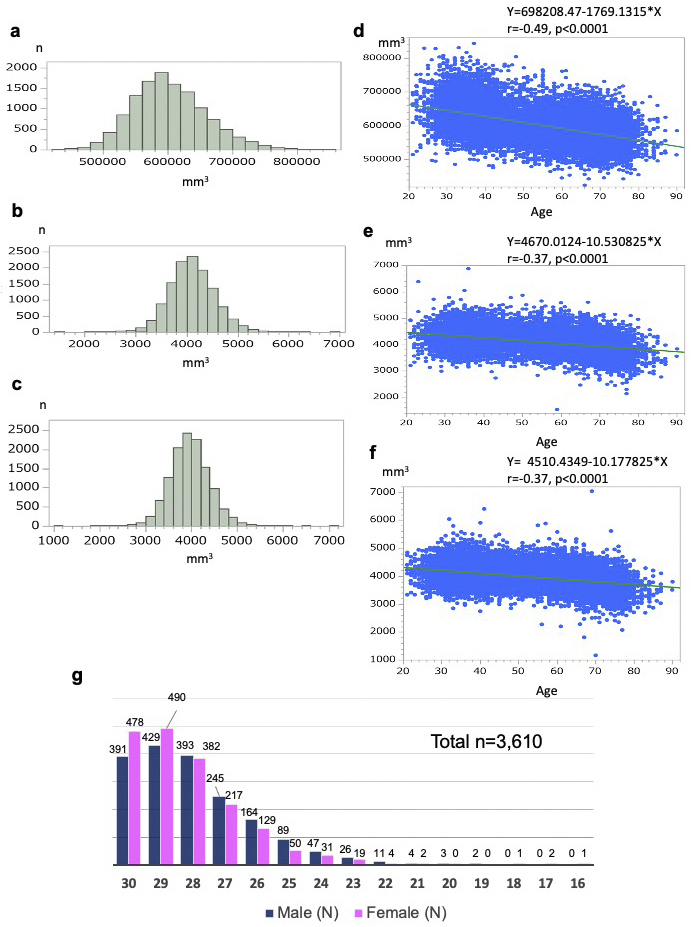
Preliminary analyses of brain morphometry and cognitive function. a-f. These figures present the distribution of anatomical volumes and the correlation between anatomical volume and age for 12,164 participants in the Brain MRI Study. a, d. Each figure presents the relationship between total gray matter volume and age. b e. Each figure presents the relationship between left hippocampal volume and age. c, f. Each figure presents the relationship between right hippocampal volume and age. Pearson’s correlation analysis was conducted to evaluate the correlation between anatomical volume and age. g. The figure presents the distribution of MMSE-J (Mini-Mental State Examination, Japanese version) scores (total and by sex), as well as the number and percentage of individuals scoring below the normal range.

First, although small-scale studies have demonstrated that brain volume is reduced with age ^(55)^, supportive evidence measuring the brain volume of the general population has not been previously reported in Japan. Because we measured brain volume using MRI scans in a population-based cohort on a large scale, we sought to elucidate how brain volume decreases with age and whether this decrease is uniform throughout the brain or differs in various regions. Thus, we examined the distributions of the gray matter volume (GMV) and the left and right hippocampus (Figure 10a-c). Furthermore, we calculated Pearson’s correlation coefficients and evaluated the correlation between anatomical volume and age (Figure 10d-f) using T1W1 data from a total of 12,164 participants. The total GMV determined by using FreeSurfer ^(52)^ (v7.2.0) data had a mean of 60.26 × 10^4^ mm^3^ and a standard deviation (SD) of 5.55 × 10^4^ mm^3^ (Figure 10a). There was a negative correlation between age and total GMV (r = − 0.49, *P* < 0.0001; Figure 10d). The left and right hippocampus had mean volumes of 4.10 × 10^3^ mm^3^ (SD = 0.43 × 10^3^ mm^3^) and 3.96 × 10^3^ mm^3^ (SD = 0.42 × 10^3^ mm^3^), respectively (Figure 10b, c). Age was negatively correlated with the left and right hippocampal volumes (r = −0.37, *P* < 0.0001 for both; Figure 10e, f). These data indicated inverse correlations between brain volume and age in many participants in the study, showing very good agreement with correlations observed in previous clinical studies ^(55)^.

Second to associate cognitive function and MRI data, we conducted a large-scale cognitive function assessment using the Mini-Mental State Examination (MMSE) ^(56)^ in a form adapted for the Japanese administration (MMSE-J) ^(57)^. The inclusion of the MMSE was principally designed to support planned research on brain changes in aging and late-life cognitive disorders. In the first (baseline) survey, the MMSE-J was administered to participants aged 65 years and above for a total of 3,610 individuals. While the majority of participants scored within the normal range, 16.2% scored below the generally accepted cutoff value of 26 points ^(56), (57)^, suggesting that these individuals may have suffered from cognitive decline (Figure 10g). Most of the suspicious scores were in the range commonly associated with mild cognitive impairment (MCI) ^(58)^, but 15 individuals scored much lower (score ≤ 21), strongly suggesting a transition to dementia. As a more sensitive assessment score than MMSE for the MCI status could then help elucidate early diagnostic markers with MRI data, the Montreal Cognitive Assessment ^(59)^, Japanese version (MoCA-J) ^(60)^, was administered to all participants in the second (first follow-up) survey.

Considering cutting-edge scientific requirements, in the second (first follow-up) survey, we introduced some changes in the imaging sequences as well as psychological and cognitive assessments (Figure 7). Given the large amount of data and its diversity, many additional analyses are planned.

## Comparison with Other Neuroimaging Studies

To the best of our knowledge, the TMM Brain MRI Study is among the largest population-based cohort studies that involve MRI worldwide. Our study has the following three salient advantages. First, all participants were genetically profiled with a Japanese-oriented microarray (Japonica-Array^Ⓡ^) ^(61), (62)^, and whole genome sequencing (WGS) ^(63)^ of these participants will be completed soon. Second, our study included participants with a bimodal age distribution, reflecting the strategic integration of a large number of elderly and young people. This age distribution enables us to conduct disease analyses focusing on each age group in isolation or populations with various ages combined. Third, the use of the North American Alzheimer’s Disease Neuroimaging Initiative (ADNI) ^(64)^ brain phantom ^(65)^ enhanced the quality of the images obtained from our study and enabled valuable cross-study data comparisons conforming to global practices.

Several recent population-based cohort studies have simultaneously acquired MRI and genome data ^(66)^, as we did. As presented in Table 4, cohort studies can consist of population- and disease-based cohort studies. Notable examples of MRI studies in the general population are the UK Biobank (UKB) ^(67)^ and our study, whereas those exclusively or primarily in patient populations include the North American ADNI ^(64)^, Japanese ADNI (J-ADNI) ^(68)^, and Enhancing Neuroimaging Genetics through Meta-analysis (ENIGMA) Consortium ^(69), (70)^. Each study has specific aims and operation protocols, but a comparison among them is intriguing.

**Table 4.** Representative Cohort Studies with Brain MRI Data.

Study	MRI Data					Non-MRI Data							
	Main purpose of brain MRI acquisition	MRI acquisition context	(Approximate) number scanned	Location/sites	Frequency/interval/maximum duration	Age range (years)	Ethnic diversity	Lifestyle/health history, PE, lab tests	Genetics	Other Brain imaging measures and relevant nonbrain imaging measures	Psychological profiling	Cognitive profiling	Linked medical records
TMM Brain MRI Study (this study)	To determine relations to dementia and other neuropsychological disorders; to support genetic and biomarker research	Addon study	12,000	Japan (one prefecture^1^, one center)	Longitudinal, Almost 5-year intervals	20-85	>99% Japanese	Yes^2^	Array (all); WGS (underway)	Retinal imaging, blood samples	Yes	Yes (older adults only)	No
UK Biobank Imaging Study ^(34), (67), (71)^, and ^(72)^	To determine relations with dementia And other neuropsychological and psychiatric disorders; to support genetic and biomarker research	Substudy	100,000	UK (entire country)	Longitudinal, variable intervals for subsets	40-69	High	Yes^3^	All	Retinal imaging, blood samples	Yes, limited	Yes (screening)	Yes
North American Alzheimer’s Diseases Neuroimaging Initiative (ADNI) ^(64)^	To understand the progression of Alzheimer’s disease	Standalone study	2424	Most US states and Canada (~60 centers)	Longitudinal, 3-12-month intervals (up to 36 months)	50+ (almost all ≥55)	Caucasian (>90%); Hispanic, Black	Yes (limited)	WGS (subset)	Amyloids and tau PET; CSF (subset, blood samples)	No	Yes (extensive AD-related)	No
Japanese ADNI (J-ADNI) ^(68)^	To understand the progression of Alzheimer’s disease	Standalone study	537	Japan (approx. 8 centers)	Longitudinal, 6-12-month intervals (up to 36 months)	60-84	>99% Japanese	Yes (limited)	Yes	Amyloids and tau PET; CSF (subset, blood samples)	No	Yes (extensive AD-related)	No
Enhancing Neuroimaging Genetics through	To assess age-related changes and the relationship to psychiatric disorders	Primary measures for most working groups	>100,000^4^	45 countries, many centers	Mixed cross-sectional/longitudinal, variable interval	0-97	Very high	Yes (extensive, content differs by project focus)	Yes	EEG, MEG, MRS	Variable: extensive for some projects	Limited	No
Meta-analysis (ENIGMA) Consortium ^(69)^, and ^(70)^													

Notes: Parenthesized numerical references correspond to bibliographic citations elsewhere in the manuscript. AD: Alzheimer’s disease; CSF: cerebrospinal fluid; EEG: electroencephalography; MEG: magnetoencephalography; MRS: magnetic resonance spectroscopy; PE: physical exam; PET: positron emission tomography; WGS: whole genome sequencing. ^1^ A prefecture in Japan is similar to a state in the USA or a shire in the UK. ^2^ Available from TMM CommCohort and TMM BirThree Cohort Study baseline visits. ^3^ More extensive for some participants than others. ^4^ Varied by participant type (no diagnosis, various psychiatric disorders)

The UKB is a country-wide, prospective, longitudinal study of approximately 500,000 participants within a relatively narrow age range (40-69 years). This database includes rich health and lifestyle information, as well as genome information ^(71), (72)^, along with MRI, cognitive assessment, and questionnaire-based psychological data. The ADNI and J-ADNI aimed to obtain data to understand Alzheimer’s disease (AD), and both studies involved brain MRI. They also collected a large number of other brain-focused biomarkers along with genome data. While our study has a wide focus and is not specific to AD research, it seems plausible that the MRI and genome data will support unique lines of research on risk factors and antecedents of symptom onset in cognitively intact adults and those with preclinical AD. Comparison of data between the J-ADNI and our study is expected to generate valuable insights into the onset and progression of AD.

Currently, a multisite, population-based, prospective cohort study ^(73)^ is underway in Japan; this study includes the diagnosis of new-onset dementia in each cohort over a long period, such as the Hisayama Study ^(74)^.

## Retrospective Limitations and Prospective Plans

The TMM Brain MRI Study has some limitations. Despite its aim to evaluate the impact of the GEJE and related tsunami in 2011, the data cannot be used to assess acute responses to the disaster. However, the study may contribute to the evaluations of the chronic impacts of the disaster on both the physical and mental conditions. In this study, we have planned and are now conducting follow-up longitudinally on the chronic health transition caused by the earthquake and tsunami through brain imaging and direct interviews in person with cognitive batteries and detailed questionnaires for adults across all generations.

Providing unique structural and functional brain MRI variables that cover both young and elderly adults enables the creation of a database that could support diverse and multimodal precision in medical research. Furthermore, the coverage of data from young generations will allow us to conduct brain-behavioral correlational studies relevant to neuropsychological disorders, such as depression and posttraumatic stress disorder, which are long-term conditions observed in the residents of the affected area.

## Conclusions

This report described the design of both the first (baseline) and second (first follow-up) survey on the TMM Brain MRI Study with preliminary findings of the first survey. The acquisition of follow-up brain MRI data, including parallel cognitive assessments, is currently underway. Our multidimensional and longitudinal study in conjunction with cohort studies are expected to contribute to the development of preventive medicine strategies for neuropsychological disorders, including age-related cognitive impairment.

## Article Information

### Conflicts of Interest

None

### Sources of Funding

The ToMMo, TMM Project, and TMM Brain MRI Study were supported, in part, by Reconstruction Agency; Ministry of Education, Culture, Sports, Science and Technology (MEXT); and the Japan Agency for Medical Research and Development [AMED, JP 21 km0105001, and JP 21 km0105002].

### Acknowledgement

We thank Drs. Naoko Minegishi and Naho Tsuchiya, Ms. Ai Etou, Tomoka Shoji, Kotomi Shingu, and Ayako Sato as well as the Tohoku Medical Megabank Project Study Group.

### Author Contributions

M.Taira, S.Mugikura, N.Mori, A.Hozawa, H.Kiyomoto, F.Nagami, Y.Taki, N.Yaegashi, H.Tomita, K.Kinoshita, S.Kuriyama, N.Fuse, and M.Yamamoto have contributed to the conceptualization or design of the study.

M.Taira, S.Mugikura, N.Mori, A.Hozawa, T.Saito, T.Nakamura, H.Kiyomoto, Tadao Kobayashi, S.Ogishima, F.Nagami, A.Uruno, R.Shimizu, Tomoko Kobayashi, J.Yasuda, S.Kure, K. Kumada, N.Nakaya, T.Obara, K.Oba, A.Sekiguchi, B.Thyreau, Y.Takano., M.Abe, N.Maikusa, Y.Taki, N.Yaegashi, H.Tomita, K.Kinoshita, S.Kuriyama, and N.Fuse have contributed to data acquisition.

M.Taira, S.Mugikura, N.Mori, T.Saito, T.Nakamura, S.Ogishima, M.Sakurai, I.N.Motoike, K.Kinoshita, and N.Fuse have contributed to data analyses.

M.Taira, S.Mugikura, N.Mori, H.Kiyomoto, J.Yasuda, T.Mutoh, Y.Tatewaki, Y.Taki, and H.Tomita have contributed to the interpretation of the study data.

M.Taira, S.Mugikura, N.Mori, A.Hozawa, Y.Taki, H.Tomita, K.Kinoshita, S.Kuriyama, N.Fuse, and M.Yamamoto have drafted the manuscript or revised it critically for important content.

M.Taira, S.Mugikura, N.Mori, A.Hozawa, Y.Taki, H.Tomita, K.Kinoshita, S.Kuriyama, N.Fuse, and M.Yamamoto have reviewed and approved the final version to be published.

M.Taira, S.Mugikura, N.Mori, A.Hozawa, H.Tomita, K.Kinoshita, S.Kuriyama, N.Fuse, and M.Yamamoto agreed to be accountable for all aspects of the work in ensuring that questions related to the accuracy or integrity of any part of the work have been appropriately investigated and resolved.

Makiko Taira, Shunji Mugikura and Naoko Mori contributed equally to this work.

### Approval by Institutional Review Board (IRB)

Institutional Review Board (IRB) approval was obtained from the Ethics Committee of the Tohoku Medical Megabank Organization (2013-4-398, latest update 2021-4-089). This study was conducted in accordance with the Declaration of Helsinki, the Ethical Guidelines for Human Genome/Gene Analysis Research, and other appropriate guidelines.
